# Novel Biological Approach to Mitigate Methane Emissions from Livestock Slurry through Microbial Conversion of Glycerol

**DOI:** 10.1021/acs.est.4c12999

**Published:** 2025-09-18

**Authors:** Herald W. Ambrose, Maria F. Bambace, Angeliki Marietou, Jiri Hosek, Anders Feilberg, Michael V.W. Kofoed, Clarissa Schwab

**Affiliations:** † Department of Biological and Chemical Engineering-Environmental Engineering, 1006 Aarhus University, Gustav Wieds Vej 10 C, DK-8000 Aarhus, Denmark; ‡ Department of Biological and Chemical Engineering-Industrial Biotechnology, 1006 Aarhus University, Gustav Wieds Vej 10 C, DK-8000 Aarhus, Denmark

**Keywords:** pig slurry, greenhouse gas emissions, methane, mitigation, *Limosilactobacillus reuteri*, glycerol, acrolein, sustainability

## Abstract

Pig farming is a major contributor to the emissions of greenhouse gases and pollutants. Methane (CH_4_), with a global warming potential 27 times that of carbon dioxide (CO_2_), accounts for up to 80% of the greenhouse gases at the farm level. Concurrently, pig production is responsible for 15% of the global livestock-related ammonia (NH_3_) emissions. Mitigation strategies that are highly effective and environmentally friendly are lacking. Here, we present a novel slurry treatment driven by the biological conversion of glycerol by *Limosilactobacillus reuteri* with the support of indigenous slurry microbiota activity, leading to the formation of the reuterin system, a broad-spectrum antimicrobial. The *in situ* production of reuterin reduced CH_4_ emissions by up to 95% in pig slurries and lowered CO_2_ and NH_3_ emissions, depending on the slurry type. Taken together, the microbial conversion of glycerol by *L. reuteri* holds promise as a biological slurry treatment to mitigate agriculture-related greenhouse and pollutant gas formation.

## Graphical abstract

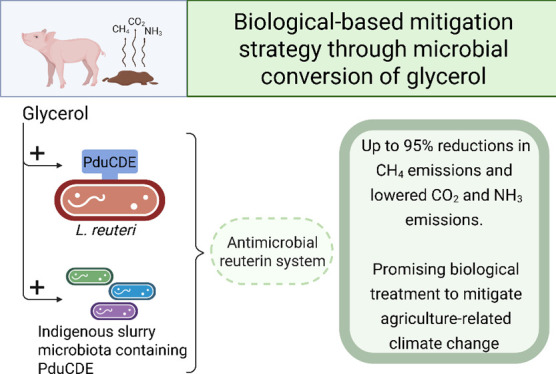

## Introduction

Global pig production has increased by 2.4-fold over the last 60 years and is expected to grow further.^1^ The growing human population and demand for meat production have led to increased livestock production worldwide^2,3^ and to a concomitant increase in greenhouse and pollutant gas emissions.^4–6^ In intensive pig production with slurry-based manure management, >90% of farmgate greenhouse gas (GHG) emissions consist of methane (CH_4_) and about 70–80% of the CH_4_ is produced during the handling and storage of slurry.^7^ This results in about 100 mt of carbon dioxide (CO_2_)-equivalent emissions released into the atmosphere globally every year, contributing to climate change and associated adverse environmental impacts. In addition, Europe emits about 3.058 kt ammonia (NH_3_) per year, and agricultural activities are responsible for 93% of anthropogenic NH_3_ emissions.^8^ Therefore, developing effective strategies to reduce microbial activity during pig slurry storage is crucial to reducing CH_4_ emissions.

CH_4_ emitted during pig manure management is the result of methanogenic microbial activity on organic substrates in fecal matter that is facilitated by the anoxic conditions below the manure surface in storage systems.^9^ Emission of CH_4_ and NH_3_ can be mitigated by physical barriers or by inhibition of bacteria and methanogen activity with the use of chemical or biological additives.^10^

Many of the previous studies on CH_4_ and NH_3_ mitigation focused on chemical additives, for example, H_2_SO_4_, and chemical inhibitors.^10^ While acidification of manure with H_2_SO_4_ is effective in reducing local emissions of CH_4_ and NH_3_, it can be considered an environmental burden due to toxicity toward terrestrial and freshwater ecosystems, and a risk to humans.^11^ High sulfur content in H_2_SO_4_-treated slurry was shown to inhibit downstream biogas production^12^ and may lower soil pH, leading to nutrient leaching upon land application.^13^

Biological strategies such as bioacidification, which is based on fermentation activity by the indigenous slurry microbiota, offer an environmentally benign solution for CH_4_ and NH_3_ mitigation from nontoxic substrates (e.g., glucose, sucrose, cheese whey).^14^ These methods reduced CH_4_ emissions of pig and cattle slurries by 40–80% during storage but required the addition of large volumes of high C/N-rich organic waste or side streams.^10,15^ Hence, novel and innovative approaches, with high CH_4_ mitigation efficiency and ease of implementation, are needed as viable alternatives to current biological or chemical additives in slurry storage.

Reuterin is a broad-range antimicrobial agent with activity against both prokaryotic and eukaryotic microorganisms; it is formed through glycerol transformation by microbes possessing the *pdu-cbi-cob-hem* operon.^16^ The key enzyme glycerol/diol dehydratase PduCDE uses glycerol to form 3-hydroxypropanal (3-HPA) as an intermediate, while 1,3-propanediol (1,3-PD) and 3-hydroxypropionate (3-HP) are the pathway end products (Figure 1). 3-HPA degrades further to the double unsaturated reactive aldehyde acrolein, and both 3-HPA and acrolein are part of the antimicrobial reuterin system.^17^ The short-chain carboxylic acid (SCCA) 3-HP is also antimicrobial and contributed synergistically to the activity of reuterin acrolein when produced by *Limosilactobacillus reuteri*.^18^ The potential for glycerol transformation is frequent among strains of *L. reuteri*.^19^ The species *L. reuteri* is Generally Recognized as Safe (GRAS) and possess Qualified Presumption of Safety (QPS) status^20,21^ and are therefore potential candidates for application in pig slurry management as an eco-friendly mitigation alternative. The aim of this study was to investigate whether *in situ*-produced reuterin is effective in mitigating CH_4_ emissions of different pig slurries during storage, addressing a key contributor to agriculture-related climate change. While CH_4_ was the primary target, NH_3_ emissions were monitored to assess potential coeffects or trade-offs associated with the treatment.

**Figure 1:**
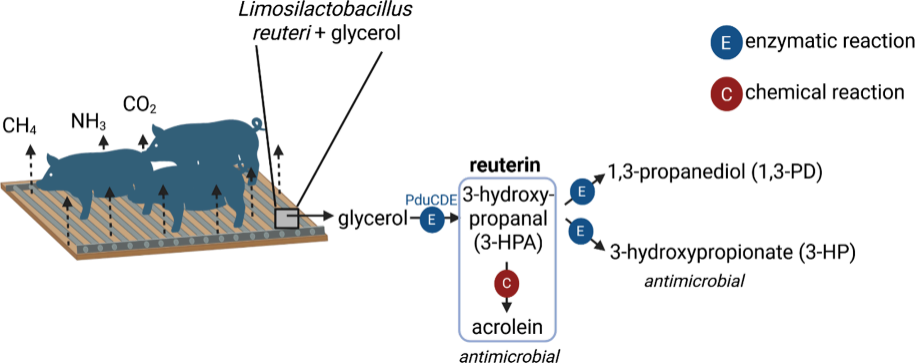
Study concept. The study used the antimicrobial reuterin system, which is produced from glycerol by a combination of enzymatic and chemical reactions. Gas emissions (CH_4_, CO_2_, and NH_3_) from the stored slurry were measured.

## Materials and Methods

### Experimental Design

Our setup simulated conditions in slurry pits as well as covered storage tanks with controlled ventilation, with an inlet air flow and an outlet flow designed for gas emission analysis. The antimicrobial effect of glycerol-transformation-based treatments on pig slurry emissions was evaluated in three sequential experiments (Figure 1 and Table 1). In experiment 1, we compared the effect of externally produced reuterin (ER) to reuterin produced *in situ* through the concurrent addition of glycerol and a strain of *L. reuteri* that possessed PduCDE. Experiment 2 assessed the effect of *in situ* reuterin production in comparison to the addition of only *L. reuteri* or glycerol as individual bioagents, while experiment 3 focused on dose optimization of *L. reuteri* and glycerol for maximum CH_4_ and NH_3_ mitigation efficiency. Each experiment included an untreated slurry and a slurry that was acidified with H_2_SO_4_ for comparison. We incubated the slurries between 25 and 30 days at room temperature (Table 1) in order to simulate storage conditions in the pig barns.

**Table 1:** Treatment Overview^a^

**Experiment**	**1**	**2**	**3**
**Slurry type**	fresh residual slurry	old residual slurry	bulk slurry
**Sampling points (days)**	0, 2, 26	0, 32	0, 2, 28
**Treatments**	untreated	untreated	untreated
	H_2_SO_4_	H_2_SO_4_	H_2_SO_4_
	externally produced reuterin (ER)	glycerol (6 g kg^–1^) (6G)	*L. reuteri* (1.2 kg g^–1^) + glycerol (3 g kg^–1^) (1.2LR+3G)
	*L. reuteri* (12 kg g^–1^) + glycerol (6 g kg^–1^) (12LR+6G)	*L. reuteri* (12 kg g^–1^) (12LR)	*L. reuteri* (12 kg g^–1^) + glycerol (3 g kg^–1^) (12LR+3G)
		*L. reuteri* (12 kg g^–1^) + glycerol (6 g kg ^–1^) (12LR+6G)	*L. reuteri* (1.2 kg g^–1^) + glycerol (6 g kg^–1^) (1.2LR+6G)
			*L. reuteri* (12 kg g^–1^) + glycerol (6 g kg^–1^) (12LR+6G)

a Three different pig slurries were stored in a headspace emission setup at room temperature to simulate storage conditions. The slurries were treated with external reuterin, *L. reuteri,* and/or glycerol at different levels, and compared to untreated slurries and slurries with H_2_SO_4_, added. Each slurry incubation experiment was run in triplicate fermentations, and samples were collected for additional analysis.

### Slurry Collection

All of the slurries were collected from different facilities housing finisher pigs (30–115 kg). For experiment 1, fresh residual pig slurry was collected from a section with a solid floor from a local farm (Spo̷ttrup, Denmark) using a manual pump. For experiment 2, residual slurry collected by scraping the slurry pits under a slatted floor at the SEGES pig research center (Gro̷nho̷j, Denmark) was used. For experiment 3, bulk slurry was manually sampled with plastic containers from a pretank located before the main storage tank at the experimental facility in Foulum, Denmark. The residual slurry collected in experiment 2 was frozen for approximately three months and thawed for experimentation. The fresh (experiment 1) and bulk slurries (experiment 3) were stored at 4 °C until experimentation. All the slurries were characterized at the start and end days of the experiments (Table S3).

#### Bacterial Strain, Culture Condition, and Production of Reuterin

*L. reuteri* SD2112 from an −80 °C glycerol stock was streaked on De Man Rogosa-Sharpe (MRS) agar plates and incubated at 37 °C for 48 h. One colony was picked and transferred to MRS broth (10 mL test tubes) containing 20 mM of glycerol and incubated at 37 °C for 24 h. To produce biomass, the grown *L. reuteri* culture was reinoculated on fresh broth (1%, 6 L final volume) and incubated at 37 °C for 24 h. Cells were collected by centrifugation at 4000 × *g* for 10 min, resuspended in 6 L of 50 mM phosphate buffer at pH 6.5, and centrifuged under the same conditions. The procedure was repeated once more. The final wet cell pellet was weighed and stored under refrigeration until addition to slurries later on the same day.

Reuterin was produced as described^19^ with slight modifications. Briefly, *L. reuteri* was incubated with 30 mL of 500 mM glycerol at 25 °C for 3 h. The reuterin-containing supernatant was separated from biomass by centrifugation at 4000 × *g* for 10 min kept under 4 °C until the treatment application the day after. Levels of glycerol, 1,3-PD, and 3-HPA were determined as outlined below.

### Slurry Treatments

In experiment 1, we evaluated the efficacy of reuterin produced *in situ* by applying 12 g kg^–1^ *L. reuteri* cell biomass and 6 mL kg^–1^ glycerol (12LR+6G) to slurry and compared it to the application of ER (Table 1). In experiment 2, we compared the impact of the addition of H_2_SO_4_ and slurry treated with only glycerol (6 g kg^–1^, 6G), *L. reuteri* (12 g kg^–1^, 12 LR) to 12LR+6G (Table 1) and untreated slurry. In experiment 3, we compared the effect of H_2_SO_4_ and different dose combinations of *L. reuteri* (1.2 and 12 g kg^–1^) and glycerol (3 and 6 g kg^–1^) to untreated slurry (Table 1). Each treatment was applied to a single bulk slurry preparation and thoroughly mixed. For *in situ* reuterin production, the slurry was treated with *L. reuteri* and mixed. Glycerol was added followed by mixing. Slurry was subsequently divided into three reactors to establish triplicate incubations per treatment. Samples were collected from the bulk slurry preparation immediately after mixing, after 2 days, and at the end of the incubation for microbiota and fermentation metabolite analysis.

### Analytical Setup and Methods

Formation of CH_4_, CO_2_, and NH_3_ during slurry storage was determined in a dynamic headspace emission setup (Figure S1), as described in detail earlier.^22^ Briefly, compressed air was continuously passed through the headspace of up to 18 parallel running reactors with a volume of 1 L. The outlets of the reactors were connected to gas analyzers through a valve system that enabled sequential sampling of headspace gases from individual reactors. While one reactor was analyzed, headspace gas from the remaining reactors was continuously purged to exhaust to prevent gas accumulation. The emissions from the reactors were analyzed with two Picarro cavity ring-down spectroscopy (CRDS) for measuring CH_4_ and CO_2_ (G2201-i, Picarro) and NH_3_ emissions (G2103, Picarro), respectively. A description of the calculations made for cumulative emissions (g kg^–1^ slurry) of CH_4_, CO_2_, and NH_3_, the emission rates (g kg^–1^ slurry min^–1^), the emission mitigation efficiency, and the natural abundance of the ^13/12^C isotope of CH_4_ can be found in the Supporting Information.

The slurry was analyzed for pH, total ammoniacal nitrogen (TAN), total nitrogen (TN), total solids (TS), and volatile solids (VS). The TAN and TN concentrations were determined using a Tveskæg Benchtop NMR sensor (NanoNord A/S). TS and VS contents were measured using the standard APHA protocol.^23^ The pH was measured by using a Portamess (Knick) pH sensor.

A 1260 Infinity II LC system with a refractive index detector (Agilent) was used to determine substrates and fermentation metabolites in slurry samples, including glycerol, 1,3-PD, propanol, 3-HPA, acetate, propionate, and butyrate, as described previously.^24^ Metabolites were extracted from 200 to 300 mg of pig slurry with 5 mM H_2_SO_4_; supernatants from batch fermentations were analyzed directly by using a Hi-Plex-H column connected to a guard column (both Agilent). The mobile phase was 5 mM H_2_SO_4_ with a flow rate of 0.6 mL min^–1^ at 40 °C. External standards were used for quantification, and the minimum detection limit was 0.01 mM. 3-HPA and 3-HP (reported as 3-HPA/3-HP) had the same retention time and were quantified based on an external standard of 3-HPA.^25^

### DNA Isolation from Pig Slurry Samples

DNA from 0.2 to 0.3 g of frozen samples was isolated using the FastDNA Spin Kit for Soil (MP Biomedicals, Germany) following the instructions. Samples were lysed at 6.0 m s^–1^ for 40 s using Lysing Matrix E tubes and a FastPrep-24 instrument (MP Biomedicals). DNA was eluted with nuclease-free water. The quality of DNA was evaluated by agarose gel electrophoresis to test for DNA degradation, and the concentration of DNA was measured with a Nanodrop.

### Quantitative PCR to Determine the Abundance of Total Bacteria, Methanogens, and Selected Species Harboring *pduC*

Quantitative PCR (qPCR) was conducted to quantify total bacterial abundance, Methanobacteriaceae and selected bacterial groups harboring pduC that were previously shown to be prevalent in intestinal microbiota, including *L. reuteri*, *Blautia obeum*, *Ruminococcus gnavus*, and *Clostridium sensu stricto* (Table 2).^26^ qPCR was run as previously described^26^ (Supporting Information).

**Table 2:** Primers Used in the qPCR Assays

**target gene**	**primer sequences (5′-3′)**	**product size (bp)**	**reference**
16S rRNA gene	F: ACTCCTACGGGAGGCAGCAG	197	^26^
	R: ATTACCGCGGCTGCTGG		
*Methanobacteriaceae* 16S rRNA gene	F: AGGAATTGGCGGGGGAGCAC	195	^27^
	R: TGGGTCTCGCTCGTTG	192	
*Limosilactobacillus reuteri* *pduC*	F: CGTTATGCACCATTCAATGCT		^26^
	R: CCATGGAGTATCATCACCATC		
*Ruminococcus gnavus* *pduC*	F: CTGAAGGTCCGCTTTACATC	370	^26^
	R: CAAACATATTGTCATAGTTCG		
*Blautia obeum* *pduC*	F: CTGAAGGTACGTTTTACCTC	370	^26^
	R: CGAACATATTGTCGTAGTTTG	370	
*Clostridium sensu stricto pduC*	F: GTR GTT GAA ATG ATG ATG	355	^26^
	R: AWG GWG TAT CRT CKC CAT		

### Microbiota Profiling with 16S rRNA Gene Sequencing and Data Analysis

Library preparation followed Illumina’s 16S Metagenomic Sequencing Library Preparation as previously described,^24^ using a two-step PCRQ, and is explained in the Supporting Information.

### Statistical Analysis

All of the treatments reported in this study were conducted in triplicates. The cumulative emissions from the experiments are expressed as the mean ± standard deviation obtained from the triplicates. The statistical significance of gas measurements was determined with one-way ANOVA with a level of significance (α) of 0.05. Posthoc Tukey tests were conducted to determine the *p* values for comparison of different treatments. A paired sample *t* test was conducted to determine the significance of difference between first and last 5 day average δ^13^ *C*_CH4_ values.

Cumulative NH_3_ emissions were correlated with cumulative CO_2_ emissions and pH (at the end of experiments) from 12LR+6G treatments in all experiments using the Pearson correlation. Statistical significance (*p* values) was determined using ANOVA.

Statistical analysis of 16S rRNA gene amplicon libraries was conducted with QIIME 2 version 2023.9.1.^28^ Alpha-diversity metrics such as observed features, Faith, Chao and Shannon index, β-diversity metrics, unweighted UniFrac, and principal coordinate analysis (PCoA) were estimated using q2-diversity after samples were rarefied (subsampled without replacement) to 20.680 sequences per sample in experiment 1, to 10.594 in experiment 2, and to 13.583 in experiment 3.

## Results

### *In Situ* Reuterin Formation Reduced CH_4_ Emissions from Stored Pig Slurry

To study the effect of reuterin on CH_4_, CO_2_, and NH_3_ emissions from pig slurry, we conducted three experiments in a headspace emission setup (Figure S1). We used fresh and old residual slurries (experiments 1 and 2), which remained in the pits after periodic flushing of the bulk slurry, and a bulk slurry (experiment 3).

In experiment 1, we evaluated the efficacy of reuterin produced *in situ* (12LR+6G) and compared to the application of ER (Table 1). The ER preparation contained 401 mM 3-HPA/3-HP, 94 mM 1,3-PD, and 22 mM glycerol. After 26 d, cumulative CH_4_ emissions were 0.02 and 0.4 g kg^–1^ from H_2_SO_4_-treated slurry and 12LR+6G-treated slurry, respectively, compared to 1.6 g kg^–1^ from both untreated and ER slurry (Figure 2A and Table S1). 12LR+6G treatment reduced CH_4_ emission by 74% compared to the untreated slurry (*p* = 0.005), while H_2_SO_4_ lowered CH_4_ emissions by >95% (*p* < 0.0001). Externally produced reuterin treatment did not impact CH_4_ emissions compared with the untreated slurry. Only H_2_SO_4_ treatment lowered CO_2_ and NH_3_ emissions by 55% (*p* < 0.0001) and 93% (*p* < 0.0001), respectively (Figure 2B,C).

**Figure 2:**
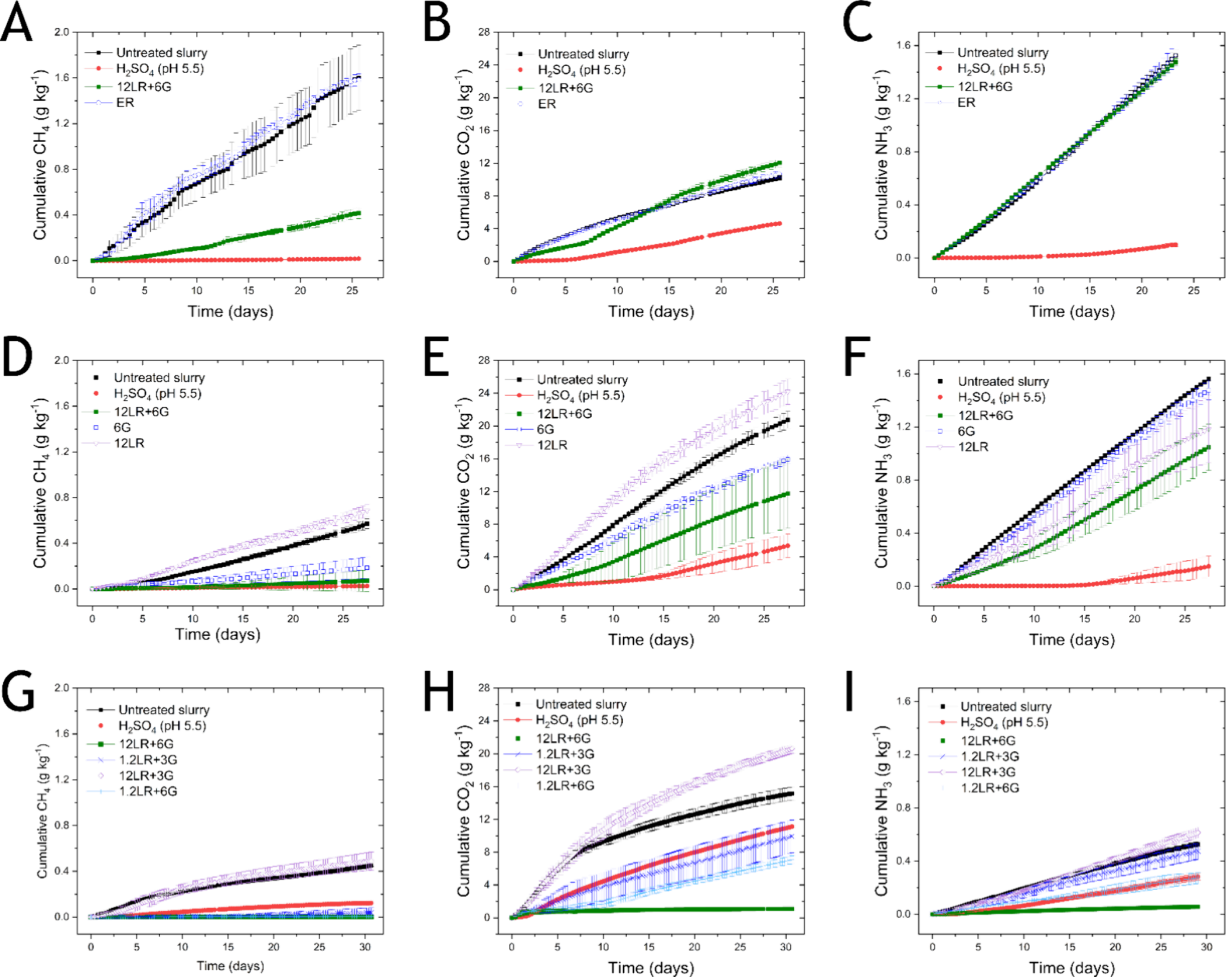
Cumulative emissions of CH_4_, CO_2_, and NH_3._ Emissions were determined during slurry storage in experiment 1 (A–C), experiment 2 (D–F), and experiment 3 (G–I) with a headspace setup and cavity ring-down spectroscopy. (A,D,G) CH_4_ emissions. (B,E,H) CO_2_ emissions. (C,F,I) NH_3_ emission. Shown are the average values from three independent incubations.

As ER did not influence CH_4_ emissions, we continued our assessment of *in situ* reuterin treatment in experiment 2 and additionally applied *L. reuteri* and glycerol separately (Table 1) to investigate the contribution of individual precursors of the reuterin system. The cumulative CH_4_ emissions from the untreated slurry were 0.57 g kg^–1^, which was less than half of the emissions observed in experiment 1 (Table S1). 12LR+6G and 6G reduced CH_4_ emissions by 88% (*p* = 0.0001) and 67% (*p* = 0.001) (Figure 2D) and lowered CO_2_ emissions by 41% (*p* = 0.01) and 23% (*p* > 0.05), respectively (Figure 2E). In contrast, 12LR led to a higher CH_4_ emission (21%). CH_4_ and CO_2_ emissions were reduced by H_2_SO_4_ (by 96 and 74%, *p* < 0.001, respectively). 12LR+6G significantly lowered NH_3_ emissions (33%, *p* < 0.05), whereas reductions in NH_3_ emissions by 12LR treatment (24%) and 6G (5%) were not significantly different (Figure 2F).

Based on the results gained in experiments 1 and 2, we optimized the dosage of *L. reuteri* and glycerol in experiment 3. We studied four combinations of *L. reuteri* and glycerol (Table 1). The cumulative CH_4_ emissions (0.45 g kg^–1^) were less than in experiments 1 and 2. 1.2LR+3G, 1.2LR+6G, and 12LR+6G reduced CH_4_ emissions by >90% (*p* < 0.0001) (Figure 2G) and reduced CO_2_ emissions by more than 30% (*p* < 0.01) with a maximum of 93% (*p* < 0.001, 12LR+6G) (Figure 2H). 12LR+3G resulted in a 6 and 36% increase in CH_4_ and CO_2_ emissions (*p* < 0.001) (Figure 2G, H). A maximum reduction in NH_3_ emissions of 89% (*p* < 0.001) was achieved with 12LR+6G, whereas 1.2LR+6G reduced emissions by 49% (*p* < 0.001) (Figure 2I). No significant reduction in NH_3_ emission was observed with 12LR+3G and 1.2LR+3G (Figure 2I). Acidification with H_2_SO_4_ lowered CH_4_ emissions by 73% (*p* < 0.0001) and reduced CO_2_ (27%, *p* = 0.01) and NH_3_ (47%, *p* < 0.0001) emissions.

Collectively, these findings demonstrate that the combination of *L. reuteri* and glycerol can reduce CH_4_ emissions by >70% while lowering CO_2_ and NH_3_ emissions depending on the slurry type. This approach presents a promising alternative to traditional acidification strategies for mitigating emissions.

### *In Situ*-Produced Reuterin Affected Methanogenic Communities in Pig Slurry

Formation of CH_4_ depends on the activity of methanogens in the stored pig slurry.^9^ To test the effect of *in situ*-formed reuterin on methanogens, we investigated community composition and abundance using 16S rRNA gene sequencing and qPCR, respectively. Concurrently, we measured the natural 13-C isotope δ^13^ *C*_CH4_ (‰) in CH_4_ emissions since δ^13^ *C*_CH4_ (‰) values provide an estimate of the dominant methanogenic pathways in the slurries during storage. Acetoclastic methanogenesis is characterized by a higher δ^13^ *C*_CH4_ (−50 to −60‰) compared to hydrogenotrophic or methylotrophic methanogenesis (−60 to −110‰).^29,30^

The untreated slurries contained three phyla (*Thermoplasmatota*, *Halobacteriota*, and *Methanobacteriota*) and six families that conduct methylotrophic (*Methanomethylophilaceae*), acetoclastic (*Methanosarcinaceae* and *Methanotrichaceae*), and hydrogenotrophic (*Methanomicrobiaceae*, *Methanocorpusculaceae*, and *Methanobacteriaceae*) methanogenesis (Figure 3A,C,E). Methanogens were initially present in a relative abundance of 0.09–0.31%. Hydrogenotrophic/methylotrophic populations represented 91–100% of the methanogenic community, and the abundance of the predominant family *Methanobacteriaceae* was 6.0–9.3 log cells g^–1^ based on qPCR (Figure 3A,C,E). Hydrogenotrophs contributed most to CH_4_ formation in stored pig slurry as indicated by an average of −77 ± 4, −92 ± 3, and −92 ± 5‰ in experiments 1, 2, and 3, respectively, during the first 5 days of storage (Figure 3B,D,F). Hydrogenotrophic methanogenesis remained predominant throughout the storage period; treatments with little effect on CH_4_ emission (ER, 12LR) followed a similar pattern.

**Figure 3:**
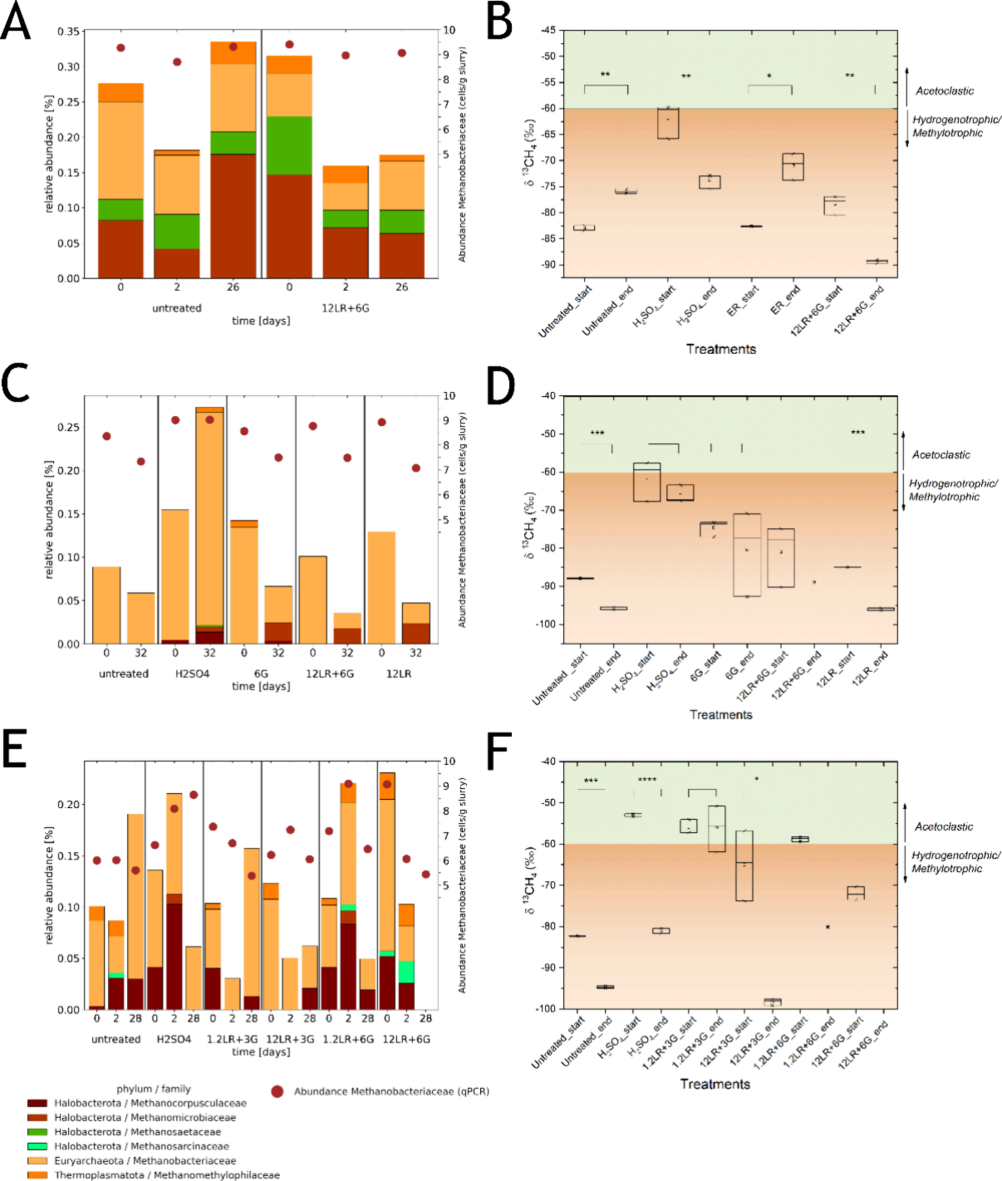
Methanogen communities and activity during slurry storage. Methanogen community composition and abundance of the predominant family *Methanobacteriaceae* were determined with 16S rRNA gene sequencing and quantitative PCR, respectively. As an indicator of active methanogen populations, we measured the natural 13-C isotope (‰) in CH_4_ emissions. (A,C,E) Methanogen community profiles and *Methanobacteriaceae* abundance in experiments 1, 2, and 3, respectively. Community profiles and *Methanobacteriaceae* abundance were not determined for the H_2_SO_4_ treatment in experiment 1. The detection limit was estimated at about 4.5 log cells g^–1^. (B,D,F) Natural 13-C isotope (‰); shown are values from the first and last 5 days of storage.

H_2_SO_4_ addition consistently lowered CH_4_ emission, while the abundance of *Methanobacteriaceae* remained stable (experiment 2, Figure 3C) or even increased by Δ2 log cell g^–1^ (experiment 3, Figure 3E). The relative abundance of *Methanobacteriaceae*, *Methanocorpusculaceae*, *Methanomicrobiaceae*, and *Methanomethylophilaceae* increased during storage of H_2_SO_4_-treated slurry. The remaining CH_4_ formation was due to the activity of hydrogenotrophs/methylotrophs (δ^13^ *C*_CH4_ around 70‰) at the end of storage. Different from H_2_SO_4_, 12LR+6G lowered cell counts of *Methanobacteriaceae* (Δ0.3–3.6 log cell g^–1^) and relative abundance of *Methanomicrobiaceae*, *Methanobacteriaceae*, and *Methanomethylophilaceae* (Figure 3A,C,E). At the end of storage, CH_4_ was mainly formed by hydrogenotrophic methanogens in experiments 1 and 2 (−88 ± 4 and −73 ± 6‰). In the 12LR+6G treatment of experiment 3, methanogens were not detected in the 16S rRNA gene data set at the end of storage, and the δ^13^ *C*_CH4_ (12LR+6G) profile could not be determined due to very low CH_4_ formation.

Together, these data show that H_2_SO_4_ reduced CH_4_ emissions without affecting abundance of methanogens, while 12LR+6G concurrently lowered methanogen abundance and activity.

### *L. reuteri* Addition Affected the Microbial Community Abundance and Diversity

As we observed divergent effects of H_2_SO_4_ and 12LR+6G on methanogenic community abundance and activity, we investigated the impact of treatment also on bacteria abundance and community composition by qPCR and 16S rRNA gene sequencing, respectively.

Bacterial cell counts in slurries were higher in experiment 1 (8.9–10.4 log cells g^–1^) than in experiment 3 (7.2–9.7 log cells g^–1^) (Figure 4A–C). While the microbiota in experiments 1 and 3 were similar in regard to richness based on Chao1 index (1056–1125), evenness (Shannon index, 6.97–7.34) and phylogenetic diversity (Faith PD 67.4–72.9), the microbiota in experiment 2 was less rich (574), even (5.4), and diverse (41.2) (Table S2). The dominant families, *Clostridiaceae* and *Peptostreptococcaceae*, accounted for 41.3 and 15.0% in the slurry of experiment 2 compared to 21.4% and 5.8–8.4% in slurries of experiments 1 and 3 (Figure 4D–F). The addition of biomass (12LR or 12LR+6G) increased the relative abundance of the *Lactobacillaceae* by 16.7–27% at the beginning of the incubation, confirming successful seeding of *L. reuteri*. In agreement, microbial communities from treatments with added biomass of *L. reuteri* (12 LR, 12LR+6G, and 12LR+3G) were distinct from unsupplemented communities in the weighted Unifrac analysis (Figure 5A,C,E).

**Figure 4:**
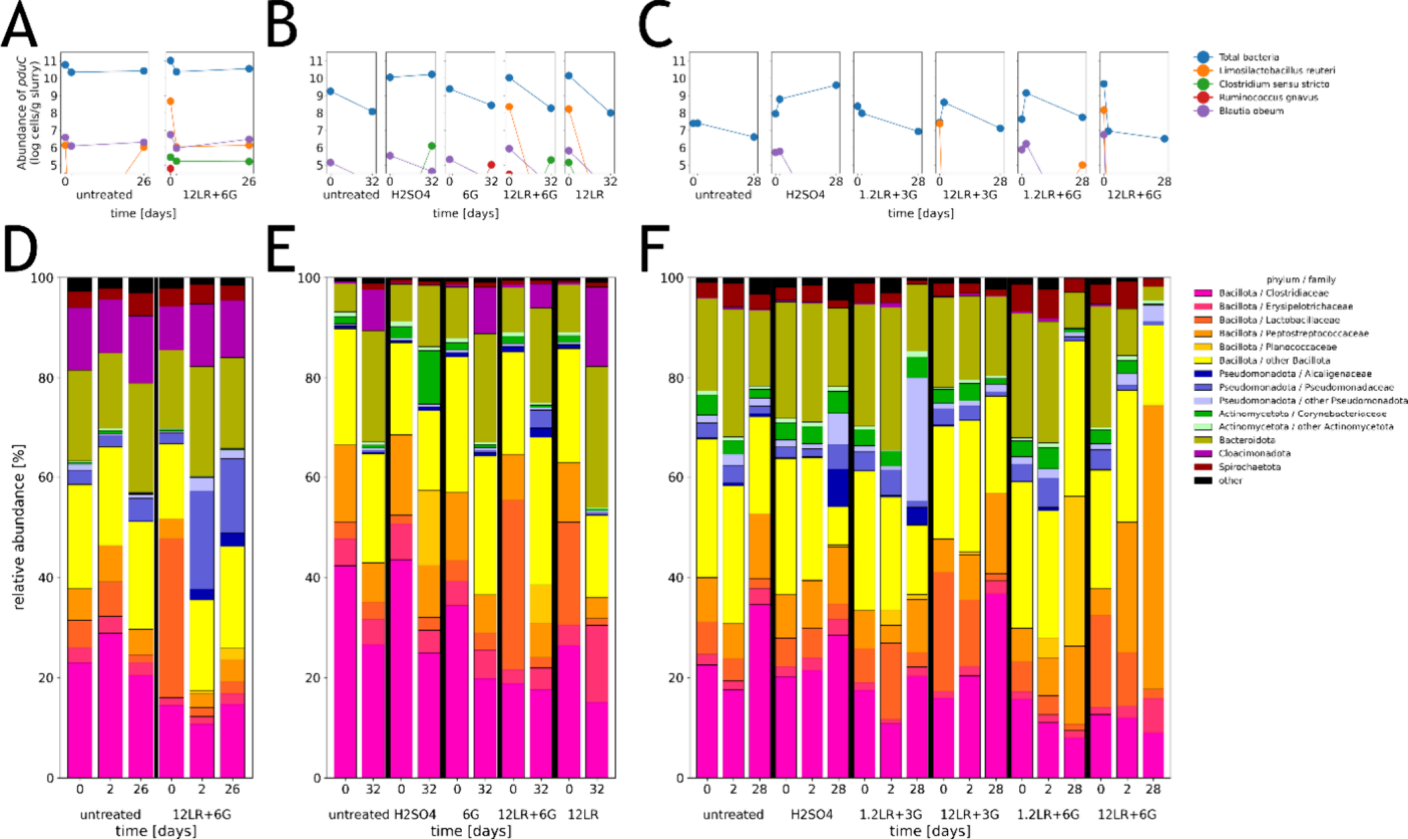
Abundance of taxa contributing to *pduC* and major bacterial families. Abundance of total bacteria and major taxa contributing *pduC* were analyzed with qPCR (A–C), while bacterial community profiles were determined using 16S rRNA gene sequencing, and (D–F) for experiment 1 (A,D), experiment 2 (B,E), and experiment 3 (C,F). The detection limit for *pduC* quantification was estimated at about 4.5 log cells g^–1^.^26^ Community profiles and *pduC* abundance were not determined for the H_2_SO_4_ treatment in experiment 1.

**Figure 5:**
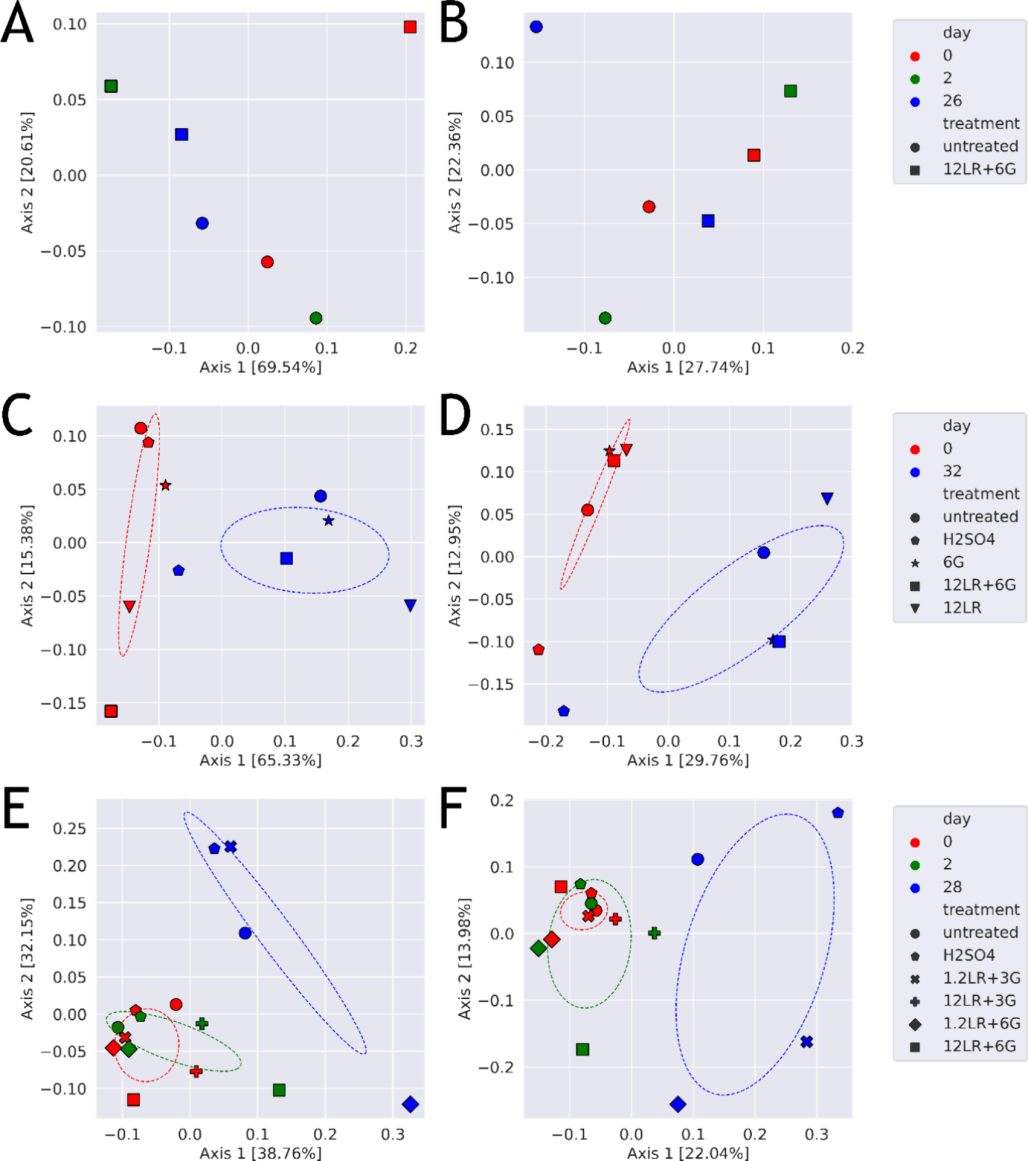
Microbial community β-diversity. Samples were collected at the beginning, on day 2, and final day of storage of experiment 1 (A,B), experiment 2 (C,D), and experiment 3 (E,F). Community composition was determined using 16S rRNA gene sequencing. Weighted (A,C,E) and unweighted (B,D,F) UniFrac plots illustrating the β-diversity of microbial communities in different pig slurries following different experimental treatments were calculated using QIIME 2.^28^ Ellipses display confidence intervals of sigma = 1.0.

By the end of the storage period, total bacterial cell counts of untreated slurries were lower than at the beginning of the experiment (Δ0.4, Δ1.1, and Δ0.8 log cells g^–1^ in experiments 1, 2, and 3, respectively), while cell counts in slurries treated with H_2_SO_4_ increased by Δ0.2–1.7 log cells g^–1^ (Figure 4A,C,E). For 12LR+6G, total cell counts decreased in experiments 1 (Δ 0.5 log cells g^–1^) and 2 (Δ 1.8 log cells g^–1^) and increased by Δ0.4 log total cells g^–1^ in experiment 3. The use of glycerol alone (6G, experiment 2) lowered cell counts by Δ0.9 log cells g^–1^ during storage (Figure 4C). For 12LR+6G treatments, α-diversity indices remained similar in experiment 1, while Faith PD, Chao1, and Shannon indexes increased in experiment 2 (14–22, 6–53, and 14–18%, respectively) and decreased in experiment 3 (9–35, 32–45, and 12–26%, respectively) (Figure 4A,C,E).

Both weighted and unweighted Unifrac analyses showed that storage time and treatment had an impact on community composition (Figure 5). After storage, *Clostridiaceae* remained as the predominant family in untreated slurries (19.1–31.9%). In slurries treated with H_2_SO_4_, the relative abundance of *Planococcaceae* (Δ14.7%) and *Corynebacteriaceae* (Δ8.3%) increased during storage in experiment 2, and of *Clostridiaceae* (Δ8.4%) and Alcaligena (Δ7.1%) in experiment 3 (Figure 4B,D,E). Treatments with combinations of *L. reuteri*/glycerol (1.2/12LR+6G) led to higher relative abundance of the *Pseudomonadaceae* (Δ12.1% versus Δ1.5%, experiment 1), *Peptostreptococcaceae* (Δ9.1–48.8% versus Δ3.6%, experiment 3), *Planococcaceae* (Δ0.1–29.1% versus Δ-0.1%, all experiments), and *Erysipelotrichaceae* (Δ0.0–5.2% versus Δ0.9%, experiment 3) compared to untreated slurry (Figure 4B,D,E).

In summary, treating the slurries with H_2_SO_4_ or *L. reuteri* and glycerol altered microbial abundance and composition in a slurry-dependent manner.

### Pig Slurries Harbored an Indigenous Population of Microbes with *pduC*

The potential for glycerol transformation by enzymes encoded by the *pdu-cbi-cob-hem* cluster is a common feature of fecal animal microbiota and a small subfraction of taxa carry the *pduC gene*.^19,26,31^ We investigated slurries for the presence of intestinal microbes harboring *pduC*, such as *L. reuteri*, *R. gnavus*, *B. obeum*, and *Clostridium sensu stricto* and used qPCR targeting the *pduC* gene as a genetic biomarker of PduCDE activity.^26^

*pduC* harboring taxa *L. reuteri*, *Clostridium sensu stricto*, *R. gnavus*, and *B. obeum* were initially detected in experiment 1 in a range of 4.3–6.8 log cells g^–1^, while *R. gnavus* (4.2–4.5 log cell g^–1^) and *B. obeum* (5.1–5.9 log cells g^–1^) were recovered in experiment 2 (Figure 4A,C,E). In experiment 3, only *B. obeum* (5.7–6.8 log cells g^–1^) was present. The addition of *L. reuteri* biomass increased the counts of *L. reuteri* to 7.4–8.7 log cells g^–1^ in treatments with the addition of 12 g kg^–1^ (Figure 4A,C,E). In general, the cell counts of the *pduC-*contributing-taxa remained stable during fermentation in experiment 1 and decreased or became undetectable in experiments 2 and 3 with the exception of *R. gnavus*, whose abundance increased when treated with H_2_SO_4_ and 6G (Δ3.8 and Δ5.0 log cell g^–1^, respectively) in experiment 2. The abundance of *Clostridium sensu stricto pduC* was higher after storage with H_2_SO_4_ and 12LR+6G (Δ6.1 and Δ5.3 log cell g^–1^, respectively) in experiment 3.

Our results revealed that potential glycerol transformers were already present in the pig slurry microbiota and that addition of *L. reuteri* successfully increased the overall potential for Pdu-driven glycerol transformation at the beginning of storage.

### Glycerol Was Utilized Immediately by Pig Slurry Microbiota with a Major Impact on Biochemical Characteristics

To determine the influence of glycerol addition and *in situ* reuterin production on microbial fermentation and the overall biochemical properties of the slurries, we monitored pH, solid content, the presence of nitrogenous compounds, and levels of SCCA as indicators of microbial fermentation activity and the compounds produced by glycerol transformation.

The initial pH values of slurries ranged from 6.8 to 7.6 and were set to 5.5 in H_2_SO_4_ treatment (Figure 6). In nonacidifying treatments, the pH value at the end of storage was similar to the untreated slurries (7.8–8.7), with the exception of 1.2/12LR+6G-treated slurries in experiment 3, which had a lower pH similar to H_2_SO_4_ (6.9–7).

The content of VS ranged from 3.4 to 5.9% in experiments 1 and 3 and from 7.4 to 8.4% in experiment 2 (Table S3). The TS and VS contents decreased in all treatments in experiment 1 except 12LR+6G, while an increase was observed in all treatments in experiment 2. %TS and %VS in experiment 3 increased or remained unchanged in all of the treatments. In all slurries, the application of 1.2/12LR+6G resulted in 1.2–1.3 times higher %VS than in untreated slurries at the end of incubation (Table S3). Initial TN and TAN were approximately 2-fold lower in experiment 3 compared to experiments 1 and 2. During incubation, TN and TAN decreased in the untreated slurry and with most treatments. The percentage of reduction was lower in experiment 3 (Table S3).

Acetate was the major SCCA (64–70% of the total SCCA), followed by propionate (20–25%), and butyrate (7–13%) (Figure 6A–C). At the end of storage, the SCCA concentrations were generally lower compared to those at the start of the experiment. SCCA levels in treatment containing 6 g kg^–1^ glycerol (1.2LR+6G and 12LR+6G) did not change during storage in experiment 3.

**Figure 6:**
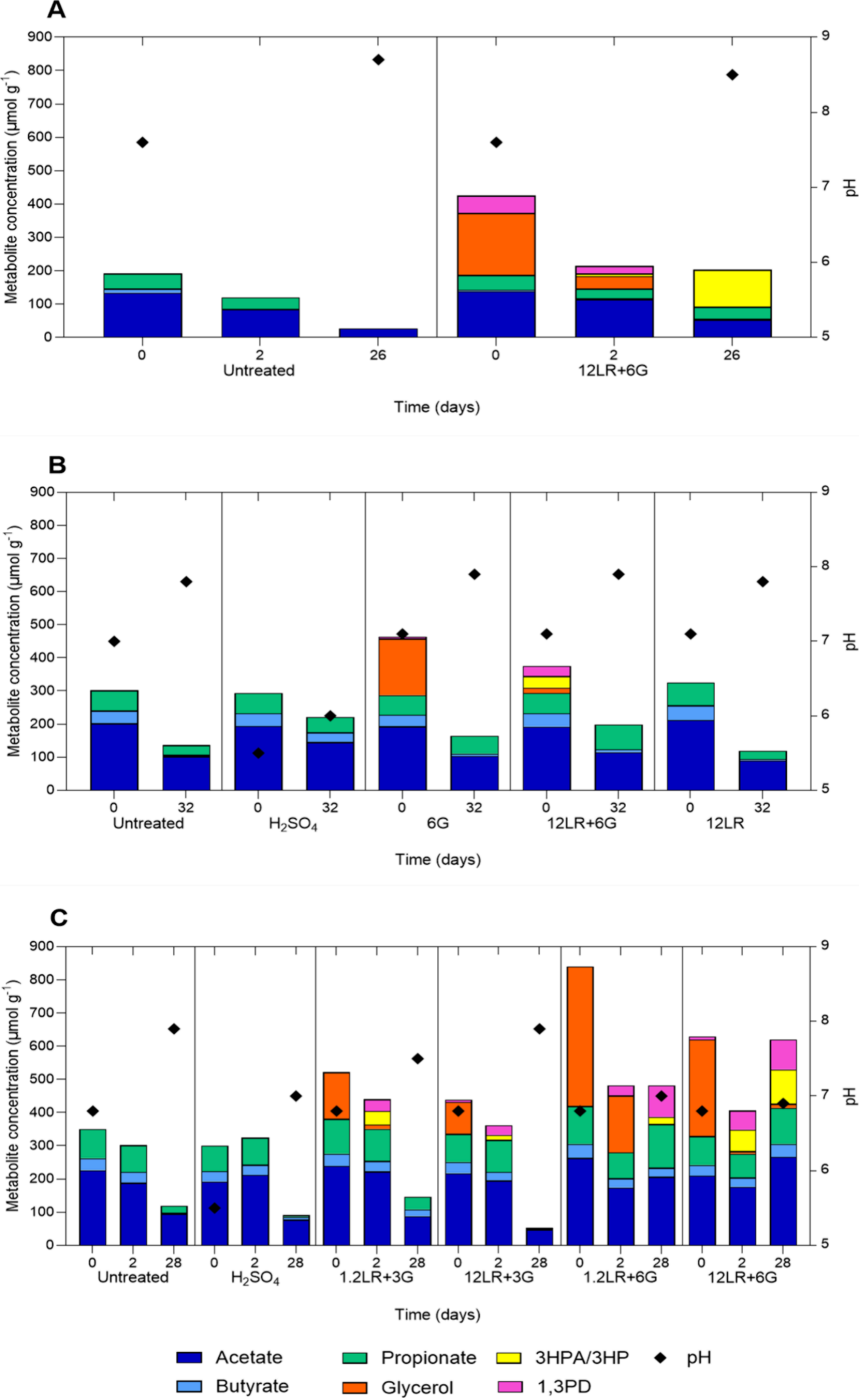
Short-chain carboxylic acid (SCCA) levels, glycerol transformation metabolites, and pH in pig slurries. SCFA, glycerol, and glycerol metabolites were analyzed at day 0, day 2, and the end of storage using HPLC-RI. Experiments 1 (A), 2 (B), and 3 (C).

Glycerol was immediately utilized when added together with *L. reuteri* biomass as indicated by the detection of 3-HPA/HP (0–35 μmol g^–1^) and 1,3-PD (0–52 μmol g^–1^) in samples from day 0 (Figure 6A–C). When glycerol was added alone (experiment 2), the transformation product 1,3-PD was detected at lower concentrations than in the concurrent 12LR+6G treatment (6 vs 30 μmol g^–1^ 1,3-PD), while 3-HPA/HP was only recovered in slurries with the 12LR+6G treatment (Figure 6B). At the same time, the treatment with higher biomass (12LR+6G) in experiment 3 led to faster glycerol utilization (8 vs 171 μmol g^–1^) and 3-HPA/3-HP (65 vs 0 μmol g^–1^) and 1,3-PD formation (57 vs 31 μmol g^–1^) compared to 1.2LR+6G as shown at day 2 (Figure 6C). At the end of experiment 3, glycerol derivatives were detected in treatments 1.2LR+6G and 12LR+6G (22–104 and 91–95 μmol g^–1^ 3-HPA/HP and 1,3-PD, respectively) (Figure 6C).

Our observations suggest that *L. reuteri* addition led to immediate glycerol transformation, and that *in situ* reuterin formation was capable of reducing microbial slurry fermentation activity, especially in the bulk slurry in experiment 3.

## Discussion

### *In Situ*-Produced Reuterin Is More Efficient in Reducing CH_4_ Emissions than Current Biological Methods

In this study, we demonstrate that application of *L. reuteri* and glycerol stored in pig slurry can effectively reduce emissions of CH_4_ in slurries from different sources with different ages and dry matter content. Previous methods of biological treatment employed variable microbial formulations with the intent to inhibit the growth of slurry microbiota through substrate competition or enhance the overall biological degradation of organic substances. For example, a mixture of microorganisms (*Rhodopseudomonas palustris*, *Bacillus* spp, *Nitrosomona europea*, *Nictobacter winogradaskyi*) (0.4 kg m^–3^) reduced CH_4_ by 8% during pig slurry storage,^32^ while “Effective Microorganisms,” a combination of lactic acid and photosynthetic bacteria, yeast, *Streptomyces* spp., and filamentous fungi showed divergent results in previous studies. Bastami et al.^33^ reported a 15–27% reduction in CH_4_ emissions from cattle slurry, while Amon et al.^34^ observed no effect. Other biological treatments relied on fermentative activity leading to acidification.^35^ Our innovative approach combined a functional microbe with a specific substrate to induce the *in situ* formation of a broad-range antimicrobial agent, achieving over 70% CH_4_ mitigation efficiency in different types of slurry. This method offers a reliable alternative to acidification but with added benefits, i.e., compatibility with subsequent anaerobic digestion, feasibility for residual slurry treatment in pits, and avoidance of sulfur accumulation in the fields that occurs with H_2_SO_4_-treated slurry.

### *In Situ*-Formed Reuterin Ensured Consistent and Sustained Reduction of CH_4_ Emissions

Glycerol/*L. reuteri* were only added at the beginning of the storage experiment, but there was persistent CH_4_ mitigation throughout the 4 weeks of incubation even though *L. reuteri* was not recovered at the end of the incubation. The most active antimicrobial component of the reuterin system is the highly reactive compound acrolein. Acrolein causes oxidative stress and can lead to DNA and protein adduct formation and enzyme inactivation at high concentrations.^17,31,36^ Due to its antimicrobial activity, acrolein is used as a biocide in a variety of industries and environments, e.g., as an aquatic herbicide.^37^

When 12LR+6G was applied in experiments 1 and 2, the pH increased during the treatment similar to that in untreated slurries, indicating that in situ-formed reuterin mitigated CH_4_ independent of pH. In contrast, the mitigation activity of H_2_SO_4_ relied on the acidification of the slurries. With *in situ* reuterin production, we introduce an alternative mitigation strategy that relies on oxidative stress caused by the formation of the reactive carbonyl species acrolein. Microbial-derived acrolein is formed by a stepwise procedure of both enzymatic (glycerol to 3-HPA) and chemical processes (3-HPA to acrolein). Conditions such as neutral pH and temperatures ranging from room temperature to 37 °C favor the conversion of 3-HPA to acrolein, which is independent of microbial activity.^17^ In general, the pH of pig slurries ranges between 7 and 8.3 ^38,39^ in agreement with our results. The common temperature in barns is about 15–25 °C,^40^ providing a suitable environment for reuterin-based treatment of pig slurries.

Our data suggest that glycerol was transformed immediately after addition and that acrolein released from glycerol supplemented at 6 g kg^–1^ was sufficient to reduce microbial activity, especially in experiment 3, as CH_4_, CO_2_, and NH_3_ formation and SCFA utilization were prevented. The reduction in CH_4_ emissions from 12LR+6G, but not from ER, suggested that *in situ* 3-HPA production and degradation provided a more sustained effect than a single addition of reuterin/3-HPA. Ramirez Garcia et al.^31^ estimated the total and bound acrolein produced in the intestinal microbiota model system and reported that after 24 h of fermentation, the majority of the released acrolein was in a bound state (>70%), suggesting that the mode of action of acrolein was a direct interaction with major cellular biomolecules in addition to a cellular oxidative stress that negatively impacted microbial cell activity.

In experiment 3, CH_4_ emissions from 12LR+3G treatment were nearly 2 orders of magnitude higher than those from 12LR+6G; emissions were even higher than the untreated slurry. The application of *L. reuteri* alone (12LR) also led to higher CH_4_ emissions compared to the untreated slurry in experiment 2. As we still observed a reduction with 1.2LR+3G, our results suggest that the ratio between added *L. reuteri* and glycerol was an important factor contributing to the antimicrobial potential of the system. When *L. reuteri* was added at 12 g kg^−1^, there can be an early limitation in the availability of glycerol when supplied at 3 g kg^−1^. In agreement, levels of glycerol transformation metabolites (3-HPA and 1,3-PD) were lower in 12LR+3G than in 12LR+6G and 1.2LR+3G. At the same time, treatments with 12LR contained more biomass, which might have been used by the slurry microbiota as nutrients, allowing for cross-feeding and CH_4_ formation. Taken together, these observations emphasize the importance of balanced dosing between *L. reuteri* and glycerol for efficient *in situ* reuterin formation.

### Effect of *In Situ* Reuterin Formation Was Similar toward NH_3_ and CO_2_

In addition to CH_4_, intensive pig production is a source of NH_3_ and CO_2_ emissions. The release of NH_3_ from manure management needs to be controlled in order to avoid the acidification or eutrophication of natural ecosystems.^41^ NH_3_ is produced through the microbial breakdown of urea by the enzyme urease, and by the decomposition of nitrogen-containing peptides and amino acids that are present in the fecal material,^6^ while CO_2_ is derived from microbial fermentation processes. The formation of NH_3_ and CO_2_ is linked, as CO_2_ emissions from manure increase the release of NH_3_ by elevating surface pH.^42^ Acidification with H_2_SO_4_ is an effective technology to mitigate NH_3_ emissions due to its impact on slurry pH, which protonates ammonia to its ionic form (NH_4_^+^) and thereby reduces its volatility.^43^ In agreement with the pH-dependent effect on NH_3_ speciation and emission, headspace levels were reduced in treatments with H_2_SO_4_.

12LR+6G treatments influenced NH_3_ emissions differently in the experiments, and the changes in NH_3_ release correlated with the effect on CO_2_ emissions (*r* = 0.92 and *p* < 0.001) and pH (*r* = 0.89 and *p* = 0.001) (Figure S2). 12LR+6G had no effect on CO_2_ and NH_3_ emissions in experiment 1 (final pH of 8.5). In slurries of experiments 2 and 3, which were characterized by lower final pH (7.9 and 6.9, respectively), 12LR+6G led to lower CO_2_ emissions and proportionally reduced NH_3_ emissions. The slurry from experiment 3 was also characterized by the lowest TN and TAN (Table S3).

Together, our observations indicate that the complete cessation of NH_3_ release in experiment 3 was due to a concurrent reduction of microbial activity by the formation of acrolein, reducing nitrogen mineralization, a comparatively small increase in pH during incubation and consequent volatilization suppression, and initial lower availability of nitrogenous compounds.

### Archaeal and Bacterial Community Composition and Metabolic Activity Were Affected Differently by H_2_SO_4_ and *In Situ*-Produced Reuterin

While slurries communities were dominated by bacteria, several families of methanogenic archaea were detected that were capable of acetogenic and hydrogenotrophic/methylotrophic methanogenesis. In our experiments, most CH_4_ was derived from hydrogenotrophic methanogenesis at the beginning of experiments in agreement with Dalby et al.,^9^ and after mitigation with H_2_SO_4_ or *in situ*-produced reuterin. Similar to Petersen et al.,^44^ we observed that H_2_SO_4_ treatment did not reduce methanogen cell counts despite the reduction in CH_4_ emission as pH was only moderately acidic.

After passage through the pig gastrointestinal tract, little degradable and fermentable carbon is present in the slurries, providing an environment with low nutrient availability. In experiments 1 and 2, the present SCCAs were partly used during incubation in all treatments, possibly to produce CO_2_. Yet, nutrient limitation was reflected in bacterial community composition, as major bacterial families were initially potential spore formers (e.g., *Clostridiaceae* and *Peptostreptococcaceae*). After treatment with both H_2_SO_4_ and 12LR+6G, a higher abundance of *Planococcaceae*)^45^ was observed possibly due to environmental stress imposed on the slurry system. Aerobes of the *Pseudomonadaceae* were higher after treatment with 12LR+6G, indicating that *in situ* reuterin formation caused changes in the oxidative status of the slurries.

### Indigenous Microbiota Contributes to Glycerol Transformation

The presence of pdu-cbi-cob-hem is a feature of animal microbiota, and *L. reuteri* that harbor PduCDE are commonly found in pigs.^19,46^ The formation of 1,3-PD in the sample treated with glycerol (where *L. reuteri* was not detected) confirmed the ability of the pig slurry microbiota to perform the glycerol transformation to 3-HPA and 1,3-PD in agreement with the presence of *pduC* from *B. obeum* and *R. gnavus*. However, glycerol metabolism by native microbiota was slower compared to the 12LR+6G treatment likely due to lower abundance of the *pduC*-harboring community than of the added *L. reuteri* biomass.

Under laboratory conditions, the molar yield of 1,3-PD from glycerol was reported to be as high as 0.76 for some strains of *Klebsiella pneumoniae* ^47^ or 0.67 for *Clostridium butyricum*,^48^ while *A. hallii* transformed glycerol to 1,3-PD at a ratio of 0.08.^49^ Particularly *L. reuteri* has been shown to secrete 3-HPA from glycerol transformation; extracellular 3-HPA accumulation depended on the presence of glucose; for a molar ratio of glucose:glycerol below 0.33, 3-HPA accumulated in yields ranging from 0.56 to 0.85.^50,51^ Pig slurries do not contain fermentable sugars; however, part of the 3-HPA was further metabolized, as indicated by the detection of 1,3-PD possibly due a variety of taxa contributing PduCDE activity. A shared functional trait, such as PduCDE activity, can thus contribute to the functional robustness of a microbial system.

### Feasibility and Implementation Potential of *L. reuteri* and Glycerol Treatments

The use of *L. reuteri* and glycerol for *in situ* reuterin production represents a biologically driven alternative to chemical inhibitors to reduce greenhouse and pollutant gas emissions from pig slurry. Glycerol, a byproduct of biodiesel production, is abundantly available, with global crude glycerol production projected to reach 6.3 million tons by 2025, including 680,000 tons from waste-based biodiesel feedstocks.^52^ Due to oversupply and limited market demand, novel methods to valorize glycerol are under active development.^52^ *L. reuteri* is currently produced food-grade, and bioprocesses to deliver *L. reuteri* cost-effectively at high density in combination with an effective downstream process to allow for immediate application at the farm level have to be developed. In our study, glycerol was also metabolized by native slurry microbes. If bioaugmentation with *L. reuteri* is not economically feasible, strategies to only add glycerol can be developed. This study is, however, limited by the variation in slurry origin, indicating the need for more standardized conditions and systematic testing of the 12LR+6G dosage across different slurry types to support field applications.

Compared to H_2_SO_4_, the approach based on *L. reuteri* and glycerol is operationally safer, as it avoids the handling of corrosive chemicals and the need for specialized acid-resistant equipment or safety precautions. However, process stability requires attention to dosing ratios, microbial viability during storage, and temperature conditions that support reuterin production. Developing adaptive dosing protocols will enhance feasibility.

The fate of reuterin byproducts such as acrolein and 3-HPA should be further evaluated in relation to anaerobic digestion and release in the environment. While this study showed CH_4_ suppression during storage, long-term effects on biogas yield and digester microbiota need validation. Acrolein degrades rapidly with a half-life of 7–25 h in water,^53^ but its volatility and potential ecotoxicity in slurry systems require further assessment before field-scale deployment.

## Supplementary Material



## Data Availability

The study is registered in ENA with accession number PRJEB76618.

## Notes

Methods and results of cumulative CH_4_ emissions (Table S1), alpha-diversity indices of pig slurry microbiota (Table S2), slurry characteristics (Table S3), schematic of headspace emission incubation setup and gas measurement (Figure S1), and relationship of cumulative NH_3_ emissions, cumulative CO_2_ emissions, and pH (Figure S2) (PDF)
