# Supplementary material for: Novel Biological Approach to Mitigate Methane Emissions from Livestock Slurry through Microbial Conversion of Glycerol
Source: Environ Sci Technol. 2025 Sep 18;59(38):20512–25. doi: 10.1021/acs.est.4c12999 (PMC12490011; doi:10.1021/acs.est.4c12999)
Supplement: Supplementary file 1 [file es4c12999_si_001.pdf]

Supporting Information for

A novel biological approach to mitigate methane emissions from livestock slurry through microbial conversion of glycerol

Herald W. Ambrose ,[1,2†] Maria F. Bambace,[2†] Angeliki Marietou,[2] Jiri Hosek,[2] Anders Feilberg,[1] Michael V.W. Kofoed,[2*] Clarissa Schwab[2*]

[1] Department of Biological and Chemical Engineering- Environmental Engineering, Aarhus University, Gustav Wieds Vej 10 C, DK-8000, Aarhus, Denmark.

[2] Department of Biological and Chemical Engineering- Industrial Biotechnology, Aarhus University, Gustav Wieds Vej 10 C, DK-8000, Aarhus, Denmark.

[*] mvk@bce.au.dk

[*] schwab@bce.au.dk

This document contains 9 pages, 2 figures, 3 tables, and additional information on the experimental procedures.

Calculations

The cumulative emissions (g kg^{-1} slurry) of CH_4, CO_2 and NH_3 were determined based on the following formula:

$$Cumulative\ emission = \sum_t \frac{E_t + E_{t-1}}{2} * \Delta t \qquad (1)$$

E_t and $E_{(t-1)}$ represented the emission rates (g kg^{-1} slurry min^{-1}) of the gaseous species measured at time points t (min) and t-1 (min), respectively. Δt (min) was the difference between time points t and t-1.

The emission rates (g kg^{-1} slurry min^{-1}) of gases measured in this study were calculated based on the following formula:

$$E = \frac{Q*(C - C_{bg})}{R*T} * \frac{M}{W} \qquad (2)$$

E was the emission rate (g kg^{-1} slurry min^{-1}), Q the headspace gas flow rate (L min^{-1}), C_i the measured concentration of the gas species (atm) in the outlet, $C_{(bg)}$ the concentration of gas species (atm) in the background, R the universal gas constant (L atm mol^{-1} K^{-1}), T the room temperature (K), M the molar mass of gas (g mol^{-1}) and W the mass of slurry (kg).

The emission mitigation efficiency of gases (CH_4, NH_3 and CO_2) was calculated based on the following formula:

$$Emission\ mitigation\ efficiency\ (\%) = \frac{Ecum_{Control\ reactor} - Ecum_{Treated\ reactor}}{Ecum_{Control\ reactor}} * 100 \qquad (3)$$

The natural abundance of $^{13/12}C$ isotope of CH_4 was analyzed based on the following formula:

$$\delta^{13}C_{CH4\,(analysed)} = \left(\frac{^{13/12}C_{sample}}{^{13/12}C_{VPDB}} - 1\right)\cdot 1000‰ \quad (4)$$

$^{13/12}C_{sample}$ and $_{13/12}C_{VPDB}$ were the measured $^{13/12}C$ isotope ratios of CH_4 in samples and reference material (13/12C ratio= 0.011802), respectively. $\delta^{13}C_{CH4\,(analysed)}$ was further corrected for $^{13/12}C$ isotope ratio of CH_4 in background according to the following equation.

$$\delta^{13}C_{CH4} = \frac{\delta^{13}C_{CH4\,(analysed)}\cdot C_{CH4} - \delta^{13}C_{CH4\,(analysed),bg}\cdot C_{CH4,bg}}{C_{CH4} - C_{CH4,bg}} \quad (5)$$

C_{CH4} was measured methane concentration (ppm) in the sample, C $_{(CH4,bg)}$ the methane concentration (ppm) in the background air, and $\delta^{13}C_{CH4}$ was the corrected $\delta^{13}C_{CH44}$ concentration (ppm) from the samples.

Quantitative PCR

A two-step qPCR protocol (40 cycles: 95 °C and 10 s, 60 °C and 30 s or three-step protocol (only for *pduC* of *Clostridium sensu stricto* 40 cycles of 95 °C and 10 s, 55 °C and 20 s, 60 °C and 30 s) followed by melting curve analysis. Each run contained negative controls without template DNA, and a tenfold dilution series of a linearized plasmid containing the target gene. To estimate cell counts, we corrected with a factor of 4.3 to account for the average number of copies of the 16S rRNA gene in the slurry microbiota and expressed as log cell count·g^{-1} slurry. A correction factor of 2.3 was used for *Methanobacteriaceae* to account for the average number

of copies of the archaeal 16S gene in the slurry microbiota. In contrast, *pduC* are single copy genes.

Microbiota profiling with 16S rRNA gene sequencing and data analysis

In the two-step PCR the V3-V4 hypervariable region of the 16S rRNA gene was amplified using Bac341F and Bac805R with adapters and a master mix containing 12.5 µL 2xKAPA HiFi HotStart readyMix (Roche) 0.5 µl forward and reverse primer, 1 µl DNA and 10.5 µl nuclease free water. PCR conditions were 25 cycles of denaturation at 95 ˚C for 30 s, annealing at 55 ˚C for 30 s and extension at 72 ˚C for 30 s followed by 72 ˚C for 5 min. The second PCR employed primers with barcode and amplification for 8 cycles. Samples were purified using Ampure XP beads (Beckman Coulter, Denmark) before sequencing. A pooled library comprising the amplicons of all samples was used for sequencing on a MiSeq sequencer (Illumina) at the Section of Microbiology at Aarhus University according to standard Illumina protocols. Negative controls included mock DNA isolation and procedure were analysed alongside samples.

Primer sequences (1) were removed using cut adapt (v4.4; -O 12 --discard-untrimmed -g TCGTCGGCAGCGTCAGATGTGTATAAGAGACAGCCTACGGGNGGCWGCAG -G GTCTCGTGGGCTCGGAGATGTGTATAAGAGACAGGACTACHVGGGTATCTAATCC --pair-adapters --minimum-length 75) (2) and only inserts that contained both primers and were at least 75 bases were kept for downstream analysis. Reads were quality filtered using the filterAndTrim function of the dada2 package (maxEE = 2, truncQ = 3, minLen = 150, trimRight = 40, trimLeft = 40). The learnErrors and dada functions were used to calculate sample inference

using pool = pseudo as parameter. Reads were merged using the mergePairs function and bimeras were removed with removeBimeraDenovo (method = pooled). Remaining Amplicon Sequence Variants (ASV) were taxonomically annotated using the IDTAXA classifier (3) in combination with the Silva v138 database (**Error! Reference source not found.**). The median number of reads per processed sample was 32,562 (range 20,680-38,226 reads) in Exp 1, 25,426.5 (range 10,594-51,823) in Exp 2 and 18,213 (range 2,311-71,956) in Exp 3, the negative control yielded four reads. One sample was removed from further analysis as it failed sequencing.

Table S1.

Cumulative CH_4 emissions from Experiments 1, 2 and 3.

**Experiment 1**	**CH_4 (g kg^{-1})**	**Experiment 2**	**CH_4 (g kg^{-1})**	**Experiment 3**	**CH_4 (g kg^{-1})**
Untreated	1.6±0.3	Untreated	0.57±0	Untreated	0.45±0.01
H_2SO_4	0.02±0	H_2SO_4	0.03 ± 0	H_2SO_4	0.12±0
ER	1.6±0.1	G	0.19±0.1	1.2LR+3G	0.04±0
12LR+6G	0.4±0	12LR+6G	0.07±0.09	12LR+3G	0.49±0.1
		LR	0.69±0.1	1.2LR+6G	0.005±0
				12LR+6G	0.003±0

Table S2.

Alpha-diversity indices of pig slurry microbiota

		Alpha diversity indices								
**Exp**	**Treatment**	**Faith PD**			**Chao1**			**Shannon**		
	**Day**	**0**	**2**	**end**	**0**	**2**	**end**	**0**	**2**	**end**
1	Untreated	73.3	70.8	72.3	1056	969	1001	7.0	6.6	7.1
	12LR+6G	66.7	67.2	65.8	926	966	926	6.6	6.7	7.1
2	Untreated	40.1	ND	47.1	574	ND	614	5.4	ND	6.4
	H_2SO_4	37.5	ND	45.0	554	ND	850	5.1	ND	5.9
	6G	48.4	ND	58.9	641	ND	933	6.0	ND	7.0
	12LR+6G	49.1	ND	54.8	812	ND	896	6.1	ND	7.0
3	Untreated	67.4	66.4	61.4	1125	961	768	7.3	7.6	6.5
	H_2SO_4	66.4	68.1	43.0	943	1029	536	7.4	7.3	6.1
	1.2LR+3G	66.0	*	42.8	985	*	554	7.6	*	6.0
	12LR+3G	57.7	55.4	*	823	776	*	7.1	7.1	*
	1.2LR+6G	77.2	75.5	48.4	1367	1534	758	8.0	8.1	5.9
	12LR+6G	72.7	60.4	*	1283	954	*	7.7	6.9	*

ND: not determined

*Samples were not included in alpha diversity analysis due to low overall read count.

Table S3.

Slurry characteristics.

	Slurry parameters									
	pH		Total solids %		Volatile solids %		TN (g l^{-1})		TAN (g l^{-1})	
	Start	End	Start	End	Start	End	Start	End	Start	End
**Experiment 1**										
Untreated slurry	7.6	8.7	7.5	5.1	5.5	3.6	5.9	3.9	3.4	2.8
H_2SO_4	5.5	6.3	7.7	7.6	5.9	5.8	6.0	5.7	3.7	3.6
19ER	7.6	8.8	7.1	5.1	5.4	3.6	5.6	4.1	3.4	2.7
12LR+6G	7.6	8.5	5.3	6.2	4.0	4.7	3.6	3.2	3.4	2.5
**Experiment 2**										
Untreated slurry	7.0	7.8	9.3	9.6	7.4	7.5	6.2	4.6	3.5	2.6
H_2SO_4	5.5	6.0	10.7	11.0	8.4	8.6	7.2	6.5	3.7	3.6
6G	7.1	7.9	9.8	9.8	8.0	7.8	5.4	4.2	3.4	2.3
12LR+6G	7.1	7.9	9.8	11.2	7.9	9.0	5.6	5.3	3.2	2.9
12LR	7.1	7.8	9.4	9.1	7.5	7.0	6.4	4.8	3.5	2.8
**Experiment 3**										
Untreated slurry	6.8	7.9	4.4	4.5	3.4	3.3	3.2	3.1	1.8	1.6
H_2SO_4	5.5	7.0	5.0	5.0	3.7	3.7	3.3	3.5	1.7	1.8
1.2LR+3G	6.8	7.5	4.6	4.8	3.5	3.6	3.1	2.6	1.7	1.4
12LR+3G	6.8	7.9	4.6	4.8	3.5	3.5	3.1	3.0	1.8	1.8

1.2LR+6G	6.8	7.0	4.4	5.2	3.4	4.0	3.0	2.8	1.8	1.6
12LR+6G	6.8	6.9	4.8	5.2	3.8	4.1	3.1	3.0	1.7	1.6

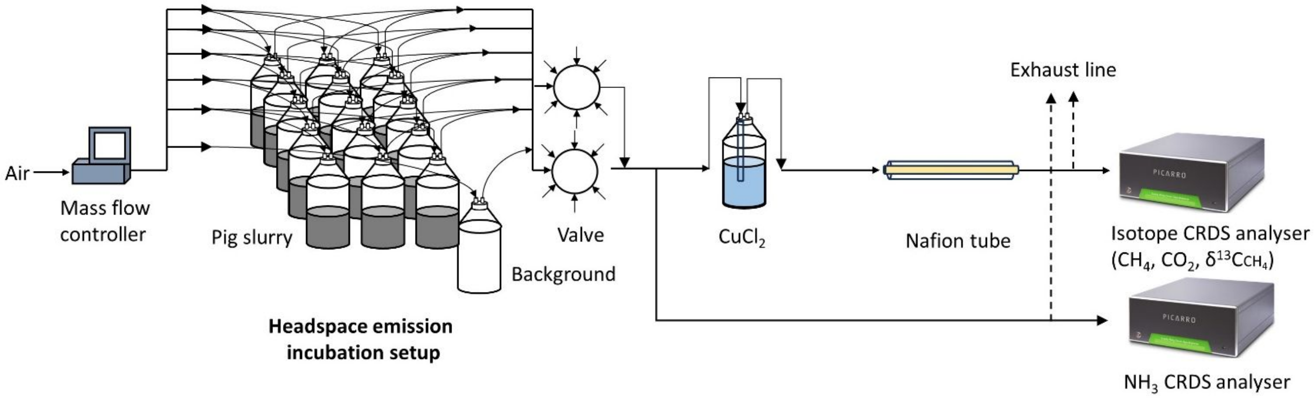

Figure S1. Schematic of headspace emission incubation set up and gas measurement.

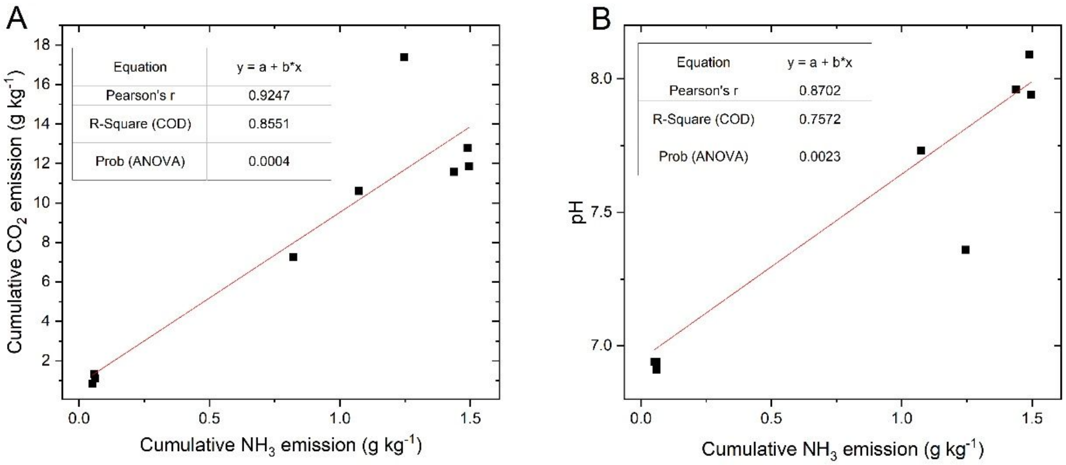

Figure S2. Relationship of cumulative NH_3 emissions and (A) cumulative CO_2 emissions and (B) pH.

References

1. K. Høgsgaard, N. P. Vidal, A. Marietou, O. G. Fiehn, Q. Li, J. Bechtner, J. Catalano, M. M. Martinez, C. Schwab, Fucose modifies short chain fatty acid and H2S formation through alterations of microbial cross-feeding activities. *FEMS Microbiol. Ecol.* **99**, fiad107 (2023).

2. M. Martin, Cutadapt removes adapter sequences from high-throughput sequencing reads. *EMBnet J* **17**,10-12 (2011).

3. A. Murali, A. Bhargava, E. S. Wright, IDTAXA: a novel approach for accurate taxonomic classification of microbiome sequences. *Microbiome* **6**, 140 (2018).

4. S. Vanderhaeghen, C. Lacroix, C. Schwab, Methanogen communities in stools of humans of different age and health status and co-occurrence with bacteria. *FEMS Microbiol. Lett.* 362, fnv092 (2015).
